# Porphyrin-Based Aluminum Metal-Organic Framework with Copper: Pre-Adsorption of Water Vapor, Dynamic and Static Sorption of Diethyl Sulfide Vapor, and Sorbent Regeneration

**DOI:** 10.3390/ma17246160

**Published:** 2024-12-17

**Authors:** Mohammad Shahwaz Ahmad, Alexander Samokhvalov

**Affiliations:** Department of Chemistry, Morgan State University, 1700 East Cold Spring Lane, Baltimore, MD 21251, USA

**Keywords:** MOF, metallo-porphyrin, diethyl sulfide, adsorption, water, controlled atmosphere, ATR-FTIR, in situ, XRD, Langmuir kinetics

## Abstract

Metal–organic frameworks (MOFs) are hybrid inorganic–organic 3D coordination polymers with metal sites and organic linkers, which are a “hot” topic in the research of sorption, separations, catalysis, sensing, and environmental remediation. In this study, we explore the molecular mechanism and kinetics of interaction of the new copper porphyrin aluminum metal–organic framework (actAl-MOF-TCPPCu) compound **4** with a vapor of the volatile organic sulfur compound (VOSC) diethyl sulfide (DES). First, compound **4** was synthesized by post-synthetic modification (PSM) of Al-MOF-TCPPH_2_ compound **2** by inserting Cu^2+^ ions into the porphyrin ring and characterized by complementary qualitative and quantitative chemical, structural, and spectroscopic analysis. Second, the interaction of compound **4** with DES vapor was analyzed dynamically by the novel method of in situ time-dependent attenuated total reflectance Fourier transform infrared (ATR-FTIR) spectroscopy at controlled humidity levels. The sorbent–adsorbate interactions, as analyzed by the shifts in IR peaks, indicate that the bonding includes the hydroxy O-H, carboxylate COO^−^, and phenyl groups. The kinetics of sorption obeys the Langmuir pseudo-first-order rate law. The pre-adsorption of water vapor by compound **4** at the controlled relative humidity under static (equilibrium) conditions yields the binary stoichiometric adsorption complex (Al-MOF-TCPPCu)_1.0_(H_2_O)_8.0_. The pre-adsorption of water vapor makes the subsequent sorption of DES slower, while the kinetics obey the same rate law. Then, static pre-adsorption of water vapor was followed by static sorption of DES vapor, and the ternary adsorption complex (Al-MOF-TCPPCu)_1.0_(H_2_O)_8.0_(DES)_3_._8_ was obtained. Despite the pre-adsorption of significant amounts of water, the binary complex adsorbs a large amount of DES: ca. 36.6 wt. % (per compound **4**). Finally, the ternary complex is facilely regenerated by gentle heating under vacuum. Compound **4** and related MOFs are promising for adsorptive removal of vapor of DES and related VOSCs from dry and humid air.

## 1. Introduction

One of the most challenging issues worldwide is environmental pollution; numerous studies have been conducted to remove environmental contaminants, understand pollution mechanisms, and develop effective solid-state functional materials for control of pollutants.

Metal–organic frameworks (MOFs) have gained significant attention as a novel class of functional solids due to their highly tunable properties that make them suitable for various applications, e.g., sensing of hazardous industrial chemicals [1], separations [2], and the sorption of toxic gases and chemical warfare agents [3].

**Figure 1:**
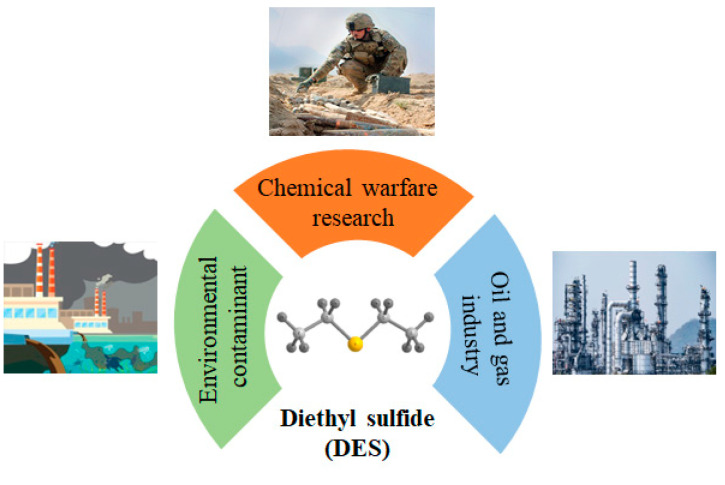
Diethyl sulfide and its presence in nature, technology, and the environment.

Volatile organic sulfur compounds (VOSCs) are significant environmental pollutants with toxicity and an unpleasant odor; they primarily originate from petroleum refining and chemical manufacturing. Moreover, VOSCs are also released to the atmosphere and water as a result of the anaerobic decomposition of organic matter [4], particularly from wastewater treatment plants, large-scale animal production facilities, and landfills [5]. The presence of these compounds in the environment is concerning due to potential impacts on both human health and ecological systems. Among the various VOSCs, diethyl sulfide (DES) is of particular interest due to its relatively simple archetypal molecular structure, which makes it representative of this class of toxic compounds (see Figure 1).

Additionally, DES is utilized in the synthesis of industrial chemicals, as a solvent, and as a simulant of the chemical warfare agent sulfur mustard [6]. There are very limited investigations on the sorption of DES by “conventional” sorbents, i.e., activated carbon [7]. The contemporary advanced sorbents notably include covalent organic frameworks (COFs), e.g., [8], and MOFs.

Among the various types of MOFs, those based on porphyrin and metallo-porphyrin linkers (also known as porphyrin MOFs) are particularly noteworthy. Their unique structural features and chemical functionalities, which are derived from porphyrins, have been studied for applications in heterogeneous photocatalysis [9], sensing, and sorption of water [10]. Certain first-row transition metal cations, when present in functional materials, have high affinity for organic compounds, notably those with carbon–sulfur and carbon–carbon bonds. For example, doping activated carbons with Ni(II) and Cu(II) yields the maximum uptakes of DES [7] as high as 440.1 and 418.7 mg/g, respectively, versus just 326.4 mg/g for doping with Cr(VI). For the development of new sorbents for pollutants in air and water, Cu(II) is more safe and environmentally friendly than carcinogenic Ni(II).

Studies of competitive behavior in sorption are of significant interest and importance. Differentiation can be made between the concurrent sorption of several adsorbates [11] and sequential sorption, where an interfering compound, such as water vapor, is pre-adsorbed before the target vapor or gas [12]. This research is particularly relevant in environmental science, chemical engineering, and applied chemistry, where the sorption of a target compound in the air occurs under varying humidity levels, which can fluctuate widely. Namely, the pre-adsorption of water vapor [12] can significantly influence the sorption efficiency of target vapor or gas. It is known that many MOFs exhibit strong adsorption of water vapor from ambient air [13]. However, to the best of our knowledge, there are no studies on the effect of pre-adsorption of water vapor on the subsequent sorption of VOSCs by any MOFs. The mechanism of sorption and bonding can be studied by complementary ex situ and in situ spectroscopic analysis. Attenuated total reflectance Fourier transform infrared (ATR-FTIR) spectroscopy is attractive for this purpose [14].

**Figure 2:**
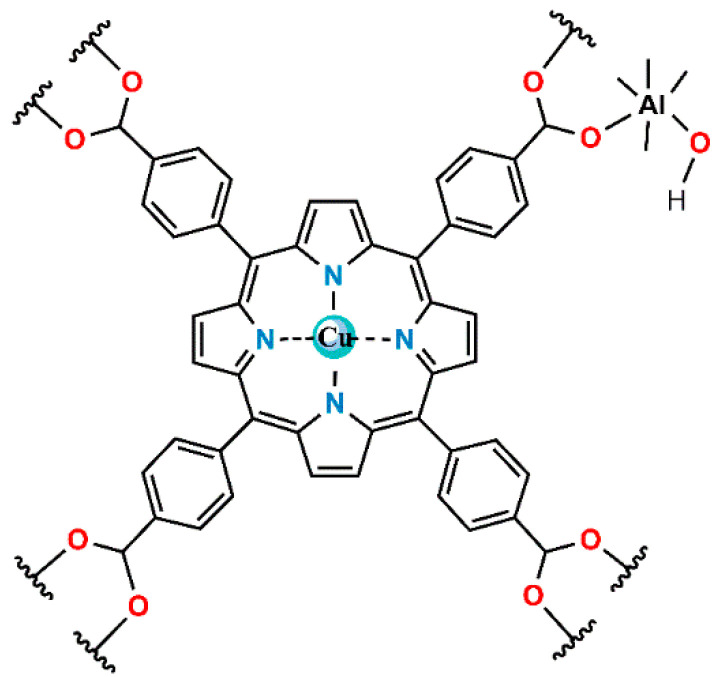
The 2D molecular structure of the structural unit of copper-containing porphyrin aluminum MOF, compound **4** actAl-MOF-TCPPCu.

We report the following:

(a) The synthesis of a new porphyrin aluminum metal–organic framework with copper in the porphyrin ring; see Figure 2 (denoted as compound **4**).
(b) The thermal activation of the product to remove the volatile impurities, followed by analysis by complementary destructive and non-destructive methods.
(c) Study of the mechanism of interaction of the activated compound **4** with vapor of DES under dynamic conditions in flowing air, using the novel method of in situ time-dependent ATR-FTIR spectroscopy in a controlled atmosphere.
(d) The pre-adsorption of water vapor on compound **4** at controlled air humidity, with the formation of the binary adsorption complex.
(e) The effect of pre-adsorbed water on the capability of compound **4** to subsequently adsorb vapor of DES under dynamic (flow) conditions.
(f) The effect of pre-adsorbed water on the ability of compound **4** to adsorb DES vapor under static (equilibrium) conditions.
(g) Facile regeneration of the so-obtained “spent” sorbent.

## 2. Materials and Methods

### 2.1. Synthesis of actAl-MOF-TCPPCu (Compound ***4***)

First, compound **2** was prepared as described by us previously [10] and shown in the top part of Figure S1.

Its chemical precursors were tetrakis(4-carboxyphenyl)porphyrin (abbreviated TCPPH_2_) with 97.0% purity (from Ambeed, Arlington Heights, IL, USA, product A114047) and aluminum chloride hexahydrate AlCl_3_·6H_2_O of 99% purity (from Thermo Scientific Chemicals, Waltham, MA, USA, product A14437.30). The solvents for the synthesis were N,N-dimethylformamide (DMF) of 99.8% min purity (from Beantown Chemical, Hudson, NH, USA, product 136300-4L) and acetone of ACS purity of 99.5+% (from Thermo Scientific Chemicals, product 030698.K2).

Then, the obtained as-prepared form of compound **2** asisAl-MOF-TCPPH_2_ was activated at 150 °C in a vacuum oven for 20 h. The vacuum oven (from Across International, Livingston, NJ, USA, model AT09e.110) was connected to a two-stage vacuum oil pump (from Xtractor Depot, Montebello, CA, USA, pumping speed 12 cfm) with base pressure < 50 mTorr as measured by the Convectron gauge and digital controller (all of Granville-Phillips, Seattle, WA, USA, model 275). This resulted in the activated form actAl-MOF-TCPPH_2_ which was stored in a jar sealed with Parafilm to prevent the sorption of humidity from air.

Next, the insertion of Cu^2+^ into the activated compound **2** was carried out using a modified procedure based on ref. [9]. Specifically, the activated compound **2** (0.1 mmol, 87 mg) and anhydrous copper acetate Cu(CH_3_COO)_2_ (0.1 mmol, 18.2 mg, ≥99.999% trace metals basis, from VWR USA, Radnor, PA, USA, product BT215040-5G) were mixed with 5 mL of DMF in a 30 mL reaction vial (from Biotage, Salem, NH, USA, product 356290). The vial with this mixture was sealed with a PTFE-lined cap and heated at 100 °C overnight in an oil bath with magnetic stirring, and then cooled to an ambient temperature. The resulting product was filtered and thoroughly washed with DMF until the filtrate was colorless.

Further, repeated washing with acetone was conducted to remove any remaining DMF. Finally, the obtained powder of asisAl-MOF-TCPPCu was activated in a vacuum at 150 °C for 20 h. This yielded the target substance actAl-MOF-TCPPCu (compound **4**), which was stored in a sealed glass jar for further use.

### 2.2. Gravimetric Analysis at Enhanced Accuracy

When operated at reduced air humidity, analytical balances are prone to weighing errors in work with electrically insulating samples due to interference with electrostatic charges [15]. To prevent this, the analytical balance of model ME204TE was equipped with a charge neutralizer (from A&D Company Americas, Ann Arbor, MI, USA, model AD-1683A). For our research on the activated MOFs, the analytical balance with a charge neutralizer was placed into a glove bag purged with dried air; each sample was briefly passed in front of the charge neutralizer, and then sample weighing was promptly conducted. For the work with pre-hydrated MOFs and MOFs saturated with DES vapor, the weighing was promptly conducted in ambient air, using the charge neutralizer as above.

### 2.3. The Quantitative Spectrophotometric Determination of Copper in Compound ***4***

First, the wet chemical digestion of compound **4** was conducted, and then the amount of Cu in the obtained solution was determined spectrophotometrically. The methodology of wet chemical digestion was developed in this work and optimized to make sure that complete dissolution of compound **4** was achieved.

Namely, 38 mg (0.04 mmol) of compound **4**, weighed using the electrostatic charge neutralizer, was added to a 25 mL round-bottom 3-neck glass digestion flask. Then, 1 mL of 70% perchloric acid HClO_4_ (from Fisher Chemical, Waltham, MA, USA, product A469-250) as the first oxidizer and acid digester was added, and the mixture was heated to boiling with a spiral reflux condenser (without water flowing through). The color of the solid sample changed to green. After this, 3 mL of 30% hydrogen peroxide H_2_O_2_ (from Thermo Scientific, product H325-500) as the second oxidizer was added dropwise for 30 min. The solution was continuously heated until it changed color from green to colorless, but some precipitate remained. After cooling, 12.5 mmol (1228 mg) of 98 wt. % sulfuric acid H_2_SO_4_ (from J.T. Baker, Phillipsburg, NJ, USA, product 9681-02) as the second acid digester was added slowly. The heating continued until the complete dissolution of the precipitate, obtaining the bluish color of Cu^2+^ in the digested liquid (non-evaporated sulfuric acid).

Then, 2 mL of deionized (DI) water was added to the flat-bottom 10 mL collection test tube; the solution from the dissolution flask was carefully transferred dropwise to water in this test tube, and the digestion flask was rinsed with 1 mL DI water which was also added to this test tube. Finally, the pH of the digestion solution in the 10 mL test tube was adjusted to pH 11.5 with concentrated ammonia solution (from J.T. Baker, USA, product 9733-01).

The amount of copper (in mg) in the obtained clear blue solution of the copper ammonia complex [Cu(NH_3_)_4_]^2+^ was determined by the UV–Visible absorbance spectroscopy [16]. Here, a dual fluorescence/absorbance spectrometer Duetta (from HORIBA Scientific, Piscataway, NJ, USA) was used with the set of Cu^2+^ standard solutions prepared to form the copper ammonia complexes. The calibration plot of the copper ammonia complex is shown in Figure S2.

To determine the mass of copper in the digested solution, the Beer law was used at the absorption wavelength of 603 nm.

To validate the method, a control experiment was conducted with the standard compound Cu(II) meso-tetra(4-carboxyphenyl)porphine of >95% purity (from Frontier Specialty Chemicals, Newark, DE, USA, CAS: 41699-93-8, product C974), abbreviated as CuTCPP. The CuTCPP standard has a molecular structure and chemical composition similar to those of compound **4**. Namely, the CuTCPP standard has a molecular weight of 852.3 g/mol and the molecular formula C_48_H_28_O_8_N_4_Cu, while compound **4** has the molecular formula C_48_H_26_O_10_N_4_Al_2_Cu and a molecular weight of 936.2 g/mol. The CuTCPP standard was digested under the same conditions and analyzed by the same method.

### 2.4. The Instrumental Characterization of Sorbent, Adsorbate, and Products of Their Interaction

FTIR spectra were obtained using an IR spectrometer (from ThermoFisher, model Nicolet iS10) in ATR-FTIR mode. This spectrometer was equipped with an ATR accessory (from Specac, Orpington, UK, model Golden Gate, part number GS10500) with a diamond ATR crystal. The data acquisition program was OMNIC version 9.2.86, and the optical aperture was set “Open”. The optical slit (corresponding to spectral resolution) was set at 4 cm^−1^ with a step of 0.48 cm^−1^. To avoid adverse effects of water vapor in the air, the FTIR spectrometer was continuously purged with dried air at a flow rate of 30 scfh (standard cubic feet per hour) as measured by the flowmeter (from Dwyer Instruments, Michigan City, IN, USA, model RMA-7). This dried air was produced by an FT-IR purge gas generator (from Parker Hannifin Corporation, Haverhill, MA, USA, model 74-5041) that provided the remaining water vapor content a with dew point of −100 °F (−73 °C) and relative humidity (RH) < 1%. This purge gas generator also removed CO_2_ to below 1 ppm. The OMNIC program had the “Atmospheric Correction” parameter enabled and the “Spectral Quality Results” parameter set at H_2_O levels ≥ 95%. The ATR-FTIR spectra were plotted in absorbance mode.

Powder X-Ray diffraction (XRD) patterns were obtained by the diffractometer model MiniFlex (from Rigaku Corporation, Tokyo, Japan). Here, a Cu K-alpha line at 0.15418 nm was used and the increments of the 2θ angle were 0.02°. This instrument had a nickel foil filter inserted to filter out the Cu K-beta artifact from the X-Rays.

The numeric peak fitting of IR spectra and XRD diffractograms was conducted using the Microcal Origin 2016 program.

### 2.5. Pre-Hydration of Compound ***4*** actAl-MOF-TCPPCu with Water Vapor

The pre-weighed activated compound **4** was placed on a watch glass in the pre-hydration chamber with controlled RH of air; this was a closed plastic desiccator where the controlled RH was created as follows.

First, controlled high humidity at RH = 75% was achieved per ref. [17] by equilibrating the air inside the closed pre-hydration chamber with a 40 mL saturated solution of sodium chloride NaCl (from Spectrum Chemical, Gardena, CA, USA, of the ACS grade, product S1240) in DI water. Continuous measurements of the RH inside the pre-hydration chamber were achieved by placing the temperature and humidity digital data logging recorder with a battery (from Elitech, San Jose, CA, USA, product GSP-8) inside it at the beginning of the experiment. The periodic observation of the RH of the air inside the pre-hydration chamber, displayed on the data logger, was performed by the in situ method, without opening the chamber, by reading the RH through a transparent lid (Figure S3).

Second, in a separate experiment, controlled medium humidity at RH ca. 55% was achieved by the same method, using a saturated solution of magnesium nitrate hexahydrate in DI water [17]. Magnesium nitrate hexahydrate Mg(NO_3_)_2_·(H_2_O)_6_ was of ACS purity with a nominal content of 98.0−102.0% (from Beantown Chemical, product 134835-500G). Third, also in a separate experiment, controlled low humidity at RH ca. 33% was achieved with a 40 mL saturated solution of magnesium chloride hexahydrate in DI water [17]. Magnesium chloride hexahydrate MgCl_2_·(H_2_O)_6_ was of ACS purity with a nominal content of 98% (from Ambeed, product A252423).

In each experiment, after an overnight period, the watch glass with the obtained sample was removed from the chamber. The obtained binary complex of compound **4** with water (also known as hydrated compound **4**) with formula (Al-MOF-TCPPCu)_1_(H_2_O)_x_ was tested in the sorption of DES vapor.

### 2.6. The Dynamic Sorption of DES Vapor by Activated Compound ***4***, and by Hydrated Compound ***4*** Using in Situ Time-Dependent ATR-FTIR Spectroscopy

The experiment was conducted using a custom-made hemispherical spectroscopic mini-chamber [18]; its advantage is a very small internal volume (a few mm^3^). Briefly, a few mg of sample was placed on the ATR crystal and covered by the mini-chamber, and the ATR screw assembly was lowered until the ATR anvil firmly pressed the specimen to the ATR crystal. The flow of dried air from the FT-IR Purge Gas Generator was decreased to a constant flow rate of 50 mL/min using a dual flowmeter (from TA Instruments, New Castle, DE, USA, part 270134.002) and then passed to the inlet of the spectroscopic mini-chamber.

First, dried air was passed through the mini-chamber, and a few ATR-FTIR spectra of compound **4** were collected. Each spectrum was averaged 64 times (96 s = ca. 1.6 min). Second, to prepare the flow of DES vapor in dried air, a facile in-flow vapor saturation setup was used [18] (a 50 mL Büchner flask about half-filled with liquid DES). The obtained stream of DES was directed to the inlet port of the spectroscopic mini-chamber, and the in situ time-dependent ATR-FTIR spectra were collected sequentially with the same settings.

The dynamic sorption of DES by the binary complex (Al-MOF-TCPPCu)_1_(H_2_O)_x_ was conducted by the same method and with the same settings.

### 2.7. The Static Sorption of DES Vapor by the Binary Adsorption Complex to the Ternary Adsorption Complex (Al-MOF-TCPPCu)_1_(H_2_O)_X_(DES)_y_ and Sorbent Regeneration

A facile organic vapor saturation chamber (a closed glass desiccator containing liquid DES) was used [18]. Immediately before inserting the sample, the interior of this organic vapor saturation chamber was purged with dried air and then a pre-weighted sample of the binary adsorption complex (Al-MOF-TCPPCu)_1_(H_2_O)_x_ was promptly inserted on the XRD sample plate. Then, the organic vapor saturation chamber was tightly closed and left overnight at room temperature. The obtained ternary adsorption complex on the XRD sample plate with nominal formula (Al-MOF-TCPPCu)_1_(H_2_O)_x_(DES)_y_ was removed from the chamber and promptly weighted to determine the y index, and its XRD pattern was collected. The regeneration of the ternary adsorption complex was conducted by heating in a vacuum oven (see Section 2.1) at 100 °C overnight. Then, the mass of the obtained sample and its XRD pattern was determined and compared to those of compound **4**.

## 3. Results and Discussion

### 3.1. Instrumental Analyses of Sorbent Compound ***4*** and Adsorbate DES

Compound **4** was prepared by post-synthetic insertion of Cu^2+^ into the TCPP ring of the porphyrin linker of compound **2**; see Figure S1.

**Figure 3:**
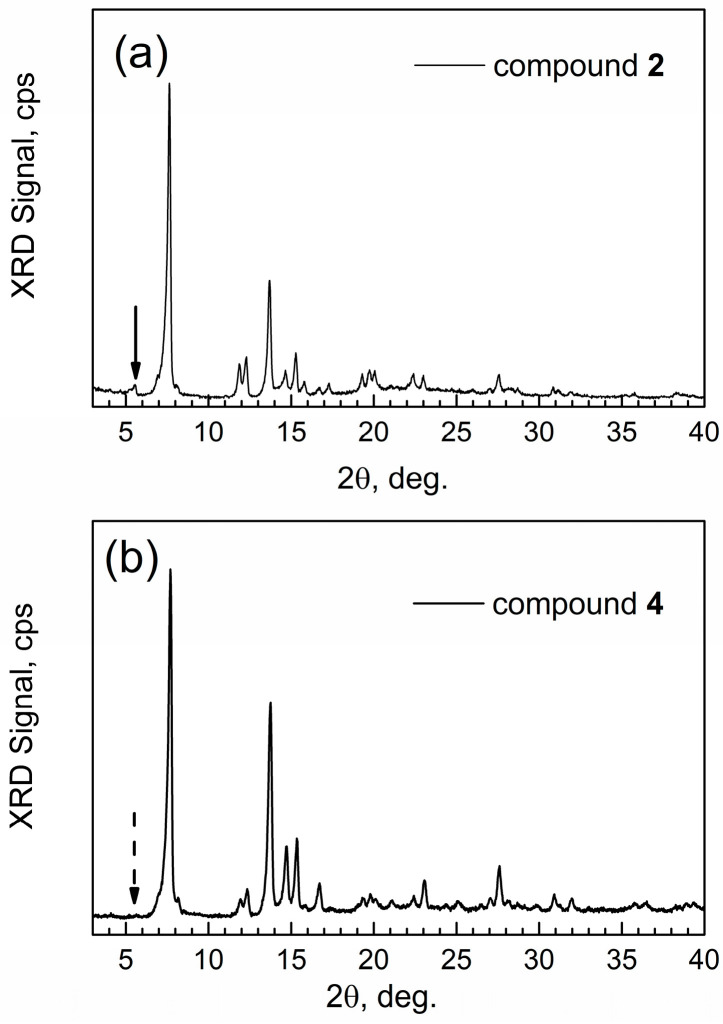
Powder XRD patterns of activated compounds: (**a**) compound **2** actAl-MOF-TCPPH_2_; (**b**) the target compound **4** actAl-MOF-TCPPCu.

Figure 3 shows the powder XRD patterns of the reactant actAl-MOF-TCPPH_2_ (compound **2**) versus the activated form of the product of this PSM reaction, actAl-MOF-TCPPCu (compound **4**). The patterns are very similar, which indicates preservation of the framework during copper insertion and the subsequent thermal activation. However, one notable change is evident: in Figure 3a, the XRD pattern of compound **2** has a small peak at ca. 5.5° (solid arrow).

In contrast, in Figure 3b, the XRD of compound **4** does not have this peak (dashed arrow) in agreement with the XRD of the similar [9] compound Al-MOF-TCPPZn; hence, the insertion of Cu^2+^ had successfully taken place. Further, for the target compound **4**, the numeric analysis of its sharp and intense peak at 2θ = 7.7° was conducted by Scherrer’s equation, D = *k* λ/β cos(θ), where D is nanocrystal size, *k* is a constant (a shape factor with numeric value 1.075 for nanoparticles [19] of spherical shape), λ is an X-Ray wavelength, β is the full-width at half-maximum (FWHM) of the peak of interest (in radian), and θ is the Bragg angle. This yields an average nanocrystal size of compound **4** at 63 nm.

It was reported that Al-MOFs have high stability in many corrosive liquids [20] and it is very difficult to digest them, even by mixtures of strong acids such as aqua regia [21]. To our knowledge, there are no reports of wet chemical digestion of any MOF containing cations of transition metals, followed by the quantitative spectrophotometric analysis of those metals. As described below, we developed and validated the procedure of wet chemical digestion of compound **4** (see Section 2). The spectrophotometric analysis of Cu in the obtained solution was conducted with its ammonia complex, and the calibration curve is shown in Figure S2. First, the validation (control) experiment by digesting the chemical standard CuTCPP and the determination of copper by the same method gives 97% nominal Cu content. Second, the same analysis of the solution after the digestion of compound **4** results in 104% of nominal Cu; hence, the PSM insertion of copper was successful.

The in situ ATR-FTIR survey spectrum of activated compound **4** provides its characteristic IR peaks (Figure S4).

The molecular structure of compound **4** is very similar to that of compound **2** except for the Cu^2+^ cation (with Cu-N bonds) in the porphyrin ring instead of the N-H bonds. The IR spectra of compound **2** were reported [10] while the vibration of the Cu-N bond is difficult to observe in IR spectra, since that peak belongs to the range of very low wavenumbers.

The major characteristic IR peaks of sorbent compound **4** are shown in Table S1.

The ATR-FTIR spectrum of DES (an adsorbate) is shown in Figure S5 and it is consistent with the literature [22].

Specifically, in Figure S5, the strongest IR peaks are (a) asymmetric CH_2_ stretching at 2965 cm^−1^, (b) combined asymmetric CH_3_ stretching and symmetric CH_2_ stretching at 2925 cm^−1^, and (c) symmetric CH_3_ stretching at approximately 2870 cm^−1^. Importantly, the IR spectrum of compound **4** (in Figure S4) does not exhibit peaks within the same range of 3000–2800 cm^−1^, and therefore, the above peaks of DES are characteristic for the study of its interaction with compound **4**.

Additionally, in Figure S5b, one can also see the deformation vibration of the CH_3_ group as a peak at 1449 cm^−1^ and the wagging vibration of the CH_2_ group as a peak at 1256 cm^−1^, and in Figure S5c, the stretch vibration of the C-C bond can be seen as a peak at 971 cm^−1^. However, these peaks are less important to our study, due to their overlap with the IR peaks of compound **4**.

In the research of the sorption of DES vapor by compound **4** by in situ time-dependent ATR-FTIR spectroscopy under a controlled atmosphere (the next section), it is effective to focus on monitoring the temporal evolution of the DES peaks within 3000–2800 cm^−1^.

### 3.2. The Progressive Sorption of DES Vapor by Activated Compound ***4*** Using in Situ Time-Dependent ATR-FTIR Spectroscopy

**Figure 4:**
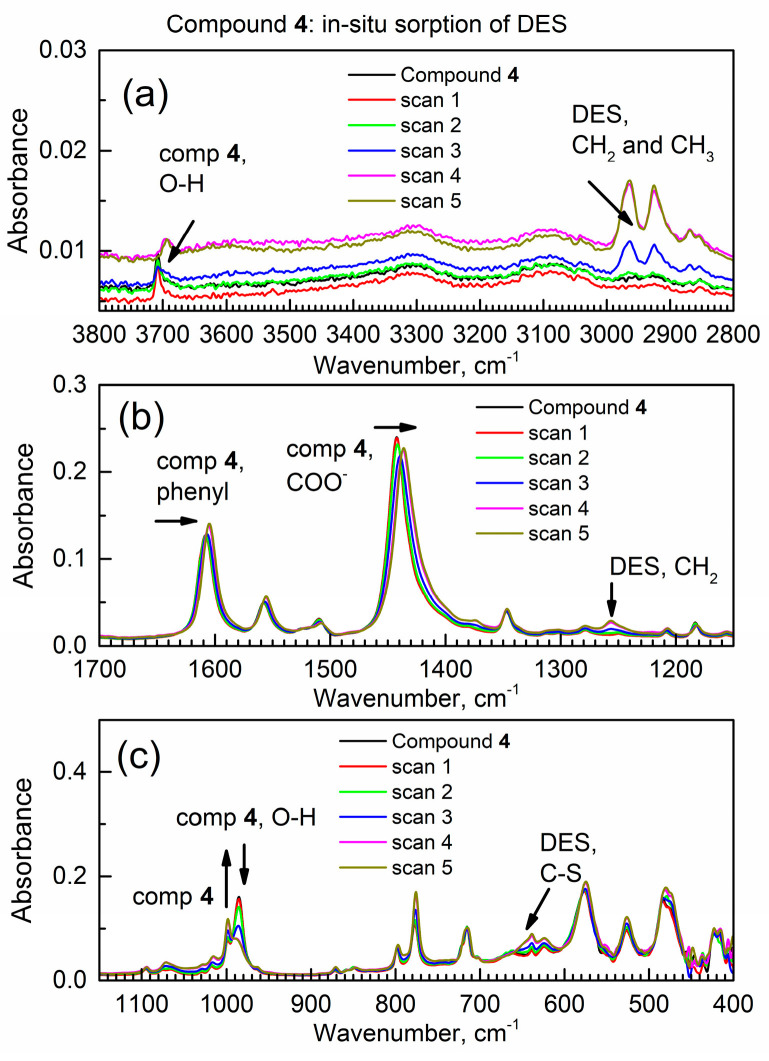
The starting in situ time-dependent ATR-FTIR spectra of compound 4 in the flow of DES vapor. (**a**) High wavenumbers; (**b**) mid-IR; (**c**) low wavenumbers.

After examining the IR spectrum of compound **4** in the in situ spectroscopic mini-chamber under purging with dried air, the gas flow was switched to DES vapor in dried air, and the first few in situ ATR-FTIR spectra of compound **4** were collected (64 scans averaged or 1.6 min per spectrum). As the time progressed, the gradual spectral IR changes were examined, as shown in Figure 4. The time-dependent changes in the ATR-FTIR spectra affect the peaks of both sorbent compound **4** and the DES adsorbate. During the subsequent 9.7−19.2 min, the in situ ATR-FTIR spectral changes are much less pronounced, and the spectra stabilize.

First, in Figure 4a, the IR peak of compound **4** at approx. 3708 cm^−1^ (the stretch vibration of the free (Al)-O-H group) exhibits a significant red shift to 3693 cm^−1^. The magnitude of this shift (Δν_1_ in cm^−1^) is significantly (a factor of approximately 4) larger than the nominal IR spectrometer resolution of 4 cm^−1^ in this experiment. The large IR peak shift Δν_1_ suggests a strong interaction between the μ(Al-O–H) group in compound **4** and DES molecules. Moreover, in Figure 4b, the two other peaks of compound **4** exhibit red shifts. The peak at ca. 1607 cm^−1^ of the phenyl group shifts from 1607 to 1604 cm^−1^ (Δν_2_ = −3 cm^−1^) (Table S2).

Additionally, in Figure 4b, the peak at ca. 1442 cm^−1^ (of the COO^−^ group) is red-shifted to 1436 cm^−1^, resulting in a shift of Δν_3_ = −6 cm^−1^. One can see the magnified (zoom) images of these spectral ranges in Figure S6. This indicates a significant noncovalent interaction between the carboxylate group of compound **4** and DES adsorbate.

The magnitude of the smallest peak shift is within the nominal resolution of the ATR-FTIR spectrometer (at optical slit at 4 cm^−1^) and significantly larger than the step size of the wavenumber (at 0.5 cm^−1^) in the spectroscopic measurements (Section 2). The relative ranking of bonding in Table S2 aligns with the strong H-bonding in the first case and weak dispersive interactions in the second case. Specifically, the lone electron pairs on the sulfur atom of DES can donate their electron density to the (Al)-O-H group of compound **4**.

Figure 4c suggests that the deformation vibration peak of the free μ(O-H) group at 985 cm^−1^ diminishes, which indicates that it is bonded with the adsorbate. This is expected based on the strong shift in the stretch vibration peak of the same group in Figure 4a. Furthermore, the adjacent peak in Figure 4c at 999 cm^−1^ increases, also supporting the formation of a bonded form, μ(O-H)····DES. Importantly, several spectral changes, namely peak shifts, absorbance increases, and absorbance decreases, only affect certain functional groups of the sorbent. This indicates that only specific groups of the sorbent interact with the adsorbate.

The dynamic changes in the DES adsorbate are also observed in Figure 4. Namely, in Figure 4a, the absorbance of all C-H stretching vibration peaks within 3000–2850 cm^−1^ increases. They are at 2965, 2925, and 2870 cm^−1^ due to asymmetric CH_2_ group stretching vibration, symmetric CH_2_ group stretching vibration combined with CH_3_ stretching, and symmetric CH_3_ stretching vibration, respectively. In Figure 4b, additionally, the growth of the peak at 1255 cm^−1^ is observed, which is attributed to CH_2_ wagging vibration. Finally, in Figure 4c, an increase in the IR peak at 648 cm^−1^ is found, which is due to the C-S bond of DES in the trans-gauche (TG) conformation [23]; also see Figure S5c for pure DES.

Figure S6 shows a zoomed view of IR peaks that experience the most significant changes.

The changes in the IR peaks of the closely located groups of the CuTCPP linker in compound **4** are shown in Figure S7.

Namely, the pyrrole and phenyl rings of the linker of compound **4** form the extended π-conjugated system of the porphyrin unit. In Figure S7a, the peak at 798 cm^−1^ is due to the breathing vibration of the pyrrole ring and the peak at 777 cm^−1^ is due to the out-of-plane CH bending mode of the porphyrin ring [24]. The red shifts in both IR peaks in Figure S7a indicate their interaction with DES, even though they are less than the shifts in the phenyl group in Figure S7b. A possible reason is that the peaks of vibrations in Figure S7a correspond to group frequencies (the IR range ca. < 900 cm^−1^) and their shifts are less pronounced due to the effect of a coupled harmonic oscillator. Namely, only a few atoms of the large molecular group interact with the adsorbate, while the wavenumber at the peak maximum is determined for the group vibration of many connected atoms.

**Figure 5:**
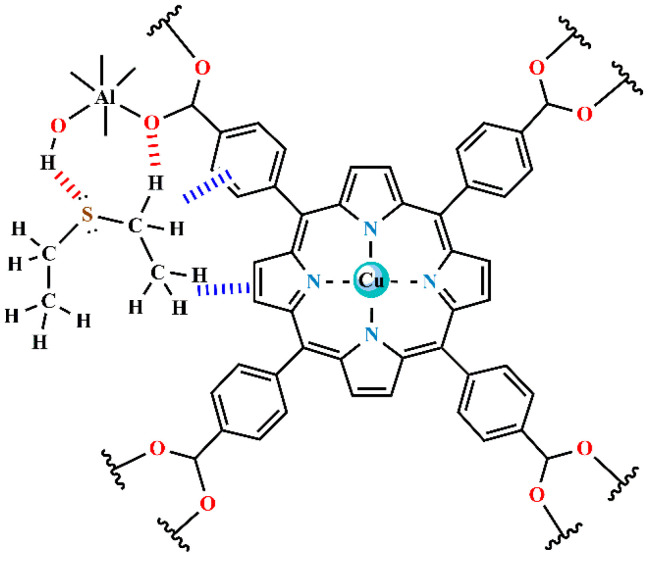
The proposed model of bonding DES molecules to compound **4**.

The sorption of DES by compound **4** proceeds by a reaction which reaches dynamic equilibrium:

Al-MOF-TCPPCu (s) + x DES (v) → (Al-MOF-TCPPCu)_1_(DES)_x_ (s) (1)

wherein (s) and (v) denote the solid and vapor phase, and (Al-MOF-TCPPCu)_1_(DES)_x_ is a solid-state product of the interaction of compound **4** with DES (the adsorption complex). Under the in situ conditions inside the mini-chamber attached to the ATR-FTIR spectrometer, one cannot conduct gravimetric analysis to obtain the stoichiometric coefficient x. The interactions of groups in compound **4** with DES are shown in Figure 5; the bonding with stronger IR peak shifts is in red, and the bonding with weaker peak shifts is in blue color.

The model of interaction can utilize the well-established sorption modalities of related inorganic solids, for example, alumina Al_2_O_3_ as a sorbent [25], which, similarly to compound **4** and related Al-MOFs, contains the surface hydroxy groups (Al)-O-H. The interaction model in Figure 5 is consistent with the bonding mechanism of related material systems [26]: the dimethyl sulfide (CH_3_-S-CH_3_) adsorbate at the surface hydroxyl groups (Al-O-H) of the alumina sorbent.

### 3.3. The Kinetics of in Situ Sorption of DES Vapor by the Activated Compound ***4***

**Figure 6:**
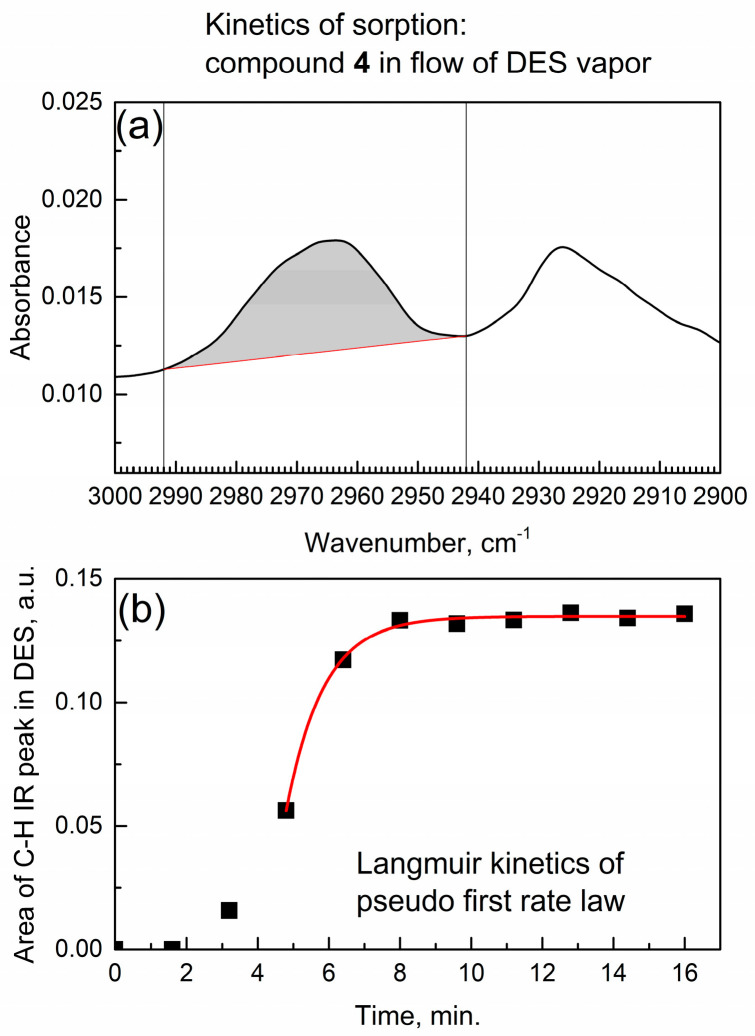
Kinetics of in situ sorption of DES vapor by the activated compound **4**. (**a**) Integration of IR peak due to the asymmetric CH_2_ stretching at 2965 cm^−1^; (**b**) kinetic analysis of the integrated peak.

To quantitatively measure the concentration of absorbed species, the integrated IR absorbance of the characteristic peaks vs. time of sorption [27] is often utilized. Figure 6a shows the typical infrared C-H peaks of DES bonded to compound **4** as observed in the in situ time-dependent IR spectrum (scan 6 at ca. 9.6 min). In Figure 6a, the IR peak of the asymmetric CH_2_ group stretching at 2965 cm^−1^ has been integrated.

In studies of kinetics of sorption, the Langmuir model is common, and the respective analytical and numeric solutions of the kinetics of sorption in gas flow are available [28]. Figure 6b shows the kinetic curve of the integrated IR peak absorbance in time. Its mathematical analysis was conducted by the Langmuir adsorption kinetics of the pseudo-first-order [29] rate law:

A_ν_(t) = B × (1 − exp(−k × t)) (2)

where A_ν_(t) is the integrated IR absorbance (area of peak with center at ν cm^−1^), B is an empirical constant, k is a kinetic rate constant, and t is time.

In Figure 6b, the initial segment of the kinetic curve (up to 4.8 min) deviates from the pseudo-first-order rate law. However, after 4.8 min, the kinetic curve was successfully fitted by adding a constant A_ν_(offset) to the formula:

A_ν_(t) = A_ν_(offset) + B × (1 − exp(−k × t)) (3)

An excellent adjusted goodness-of-fit parameter R^2^_adj_ = 0.997 was obtained and the Langmuir adsorption kinetic rate constant was determined at k(act) = 0.95 ± 0.05 min^−1^.

### 3.4. The Pre-Hydration of Compound ***4***, Followed by Dynamic Sorption of DES Vapor by in Situ Time-Dependent ATR-FTIR Spectroscopy in a Controlled Atmosphere

First, compound **4** actAl-MOF-TCPPCu was allowed to absorb water vapor in air at high RH = 75% (see Section 2). The mass increase due to water sorption is by the following reaction:

1 Al-MOF-TCPPCu (s) + 8.0 H_2_O (vap) → [Al-MOF-TCPPCu]_1.0_(H_2_O)_8.0_ (s) (4)

The product is also denoted as the binary adsorption complex, also known as compound **4hyd**. Compound **4** has the molecular Hill formula C_48_H_26_O_10_N_4_Al_2_Cu and molar weight (MW) of 936.2 mg/mmol, while the obtained binary adsorption complex has an MW = 936.2 + 8 × 18.02 = 1080.36 mg/mmol. The adsorbed amount of water corresponds to 100% × (8 × 18.02)/936.2 = 15.4 wt. %.

**Figure 7:**
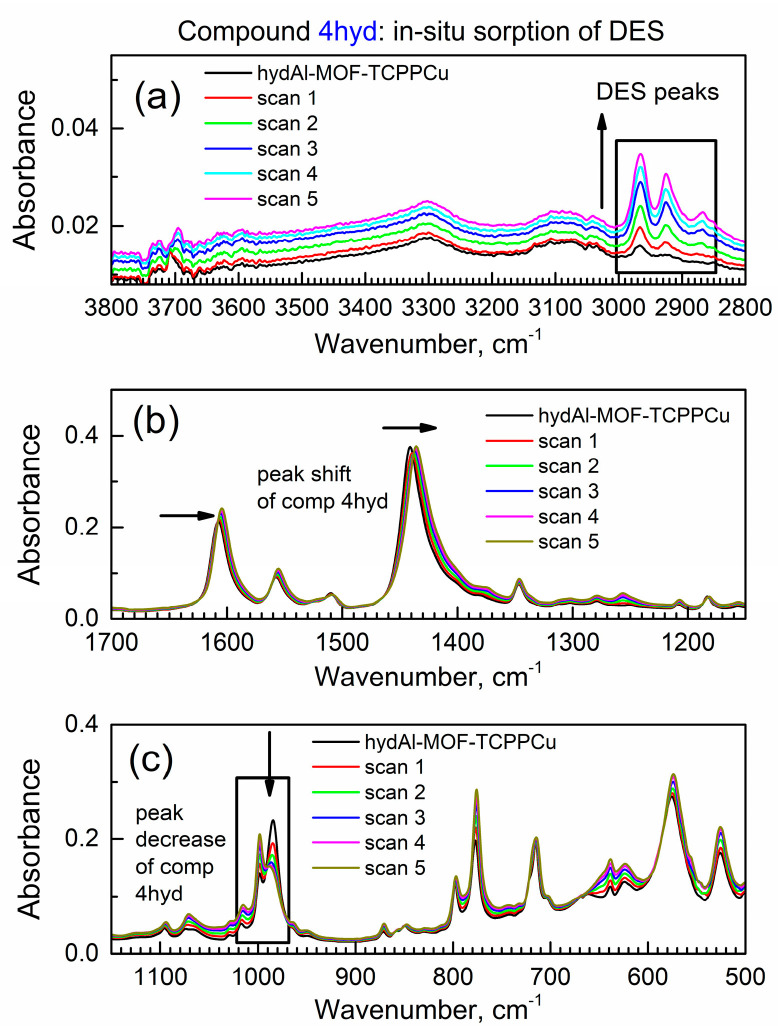
The in situ time-dependent ATR-FTIR spectra of the binary adsorption complex hydAl-MOF-TCPPCu in the flow of DES vapor. (**a**) High wavenumber range; (**b**) mid-IR range; (**c**) low wavenumber range.

Figure 7 shows the in situ time-dependent ATR-FTIR spectra of the binary adsorption complex hydAl-MOF-TCPPCu during its interaction with flowing DES vapor. In Figure 7a, the peak at ca. ν = 3708 cm^−1^ due to the stretching vibration of the (Al)-O–H group was too weak to observe due to its interaction with pre-adsorbed water. With this exception, the pattern of changes in the IR peaks during the in situ sorption is similar to that of the activated compound **4**. Hence, the binary complex adsorbs DES from the vapor phase under dynamic conditions.

Figure S8 shows the IR peaks of significant change.

In Figure S8a, growth is observed for the asymmetric (asymm.) CH_2_ stretching peak at 2965 cm^−1^ and the combination peak (asymm. stretching of CH_3_ and symmetric stretching of CH_2_) at 2925 cm^−1^ due to progressive sorption of DES by compound **4hyd**. In Figure S8b, the peak of the phenyl group is red-shifted from 1608.5 to 1604.5 cm^−1^ (with Δν = −4.0 cm^−1^). In Figure S8c, the peak at 1441.3 cm^−1^ (the COO^−^ group) is red-shifted to 1435.9 cm^−1^ with large Δν_3_ = −5.4 cm^−1^ (Table S3).

Finally, in Figure S8d, the peak of the deformation vibration μ(O-H) of the free OH group at 984.5 cm^−1^ decreases, while the peak at 998.4 cm^−1^ grows. This indicates the formation of bond μ(O-H)—DES despite the presence of a large amount of pre-adsorbed water in the sample.

**Figure 8:**
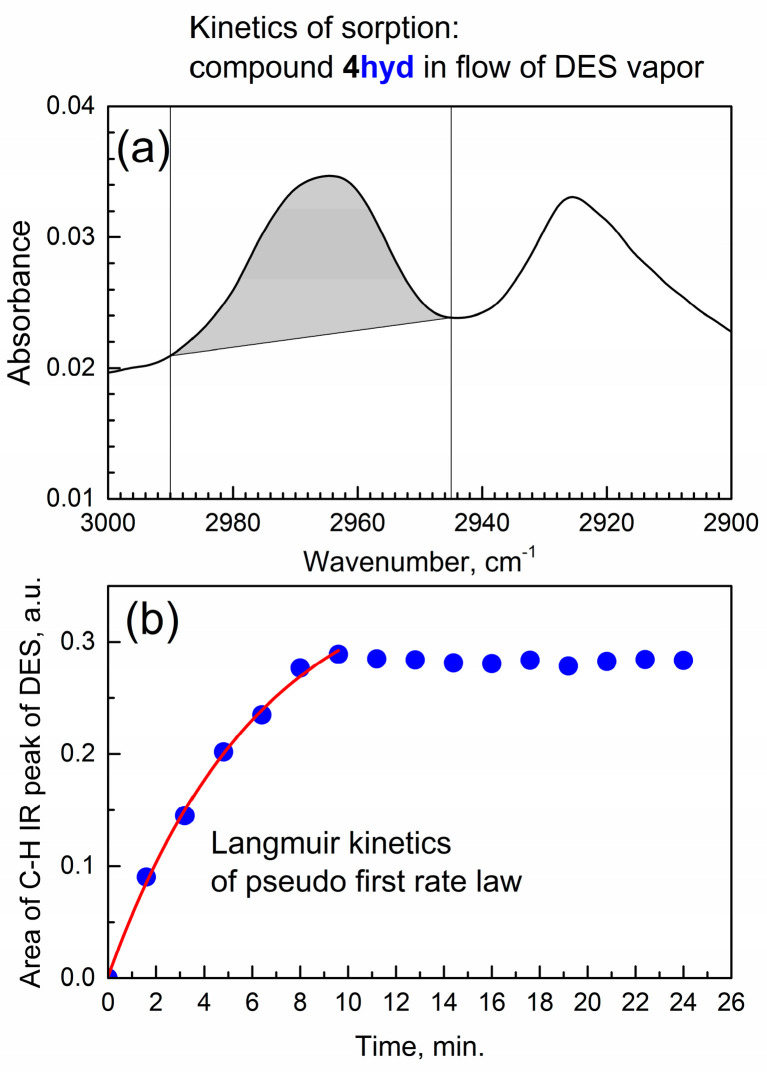
Kinetics of in situ sorption of DES vapor by compound **4hyd**. (**a**) Integrated IR peak of asymmetric CH_2_ stretching; (**b**) formal kinetic analysis of peak area in time.

Figure 8 shows the kinetics of DES sorption by the binary complex compound **4hyd**.

The same kinetic rate law is observed as for the activated compound **4**, but for the hydrated compound **4**, the corresponding curve starts from time zero; the difference is due to the effect of pre-adsorbed water. Hence, for the hydrated compound **4**, we used the standard formula [29] of the Langmuir pseudo-first-order rate law A_ν_(*t*) = B × (1 − exp(−k × *t*)). The data in Figure 8b are well fitted with this model, resulting in the rate constant k(hyd) = 0.16 ± 0.03 min^−1^ and very good R^2^_adj_ = 0.997. One can compare the k(hyd) = 0.16 ± 0.03 min^−1^ with k(act) = 0.95 ± 0.05 min^−1^; the pre-adsorption of water significantly makes slower the subsequent sorption of DES vapor. On the other hand, the sorption sites are apparently the same, based on the similar shifts in IR peaks of groups of the two sorbents (Tables S2 and S3).

For the samples of the pre-hydrated compound **4** prepared at lower RH (ca. 33% and 55%, see Section 2), no adsorbed water was observed in the ATR-FTIR spectra; hence, they were not tested in the dynamic sorption of DES vapor.

Finally, an attempt was made to regenerate the “spent” solid under the in situ conditions; the gas flow was changed to dried air and ATR-FTIR spectra were collected multiple times. The IR absorbance of the peaks of the DES adsorbate decreased but did not reach zero (no regeneration).

### 3.5. The Pre-Hydration of Compound ***4***, Followed by the Static Sorption of DES Vapor and Sorbent Regeneration

Figure S9 shows the data of gravimetric analysis of the sample after the corresponding steps.

The sorption of DES vapor by the binary adsorption complex compound **4hyd** resulted in a substantial mass increase and reaction toward the ternary adsorption complex:

(Al-MOF-TCPPCu)_1.0_(H_2_O)_8.0_ (s) + 3.8 DES (v) → 1 (Al-MOF-TCPPCu)_1.0_(H_2_O)_8.0_(DES)_3.8_ (s) (5)

The binary complex has MW = 1080.4 mg/mmol and it adsorbs DES in the amount of 3.8 × 90.2 = 342.8 mg (per one mmol of compound **4** as the porous material and active ingredient). Compound **4** has an MW = 936.2 mg/mmol; therefore, the adsorbed amount of DES is quite significant (on a gravimetric basis): 100% × 342.8 mg DES/936.2 mg compound **4** = 36.6 wt. % or 366 mg/g. The “conventional” sorbents (activated carbon impregnated with Ni(II), Cr(VI), Co(II), Cu(II) or Cd(II)) yield the maximum uptake of DES [7] at 440.1 mg/g; however, those studies were conducted without pre-adsorption of water.

Upon regeneration, the mass of the sample became the same as that of the activated compound **4**, which means complete regeneration of the ternary adsorption complex:

(Al-MOF-TCPPCu)_1.0_(H_2_O)_8.0_(DES)_3.8_ (s) → (Al-MOF-TCPPCu) (s) + 8 H_2_O (v) + 3.8 DES (v) (6)

**Figure 9:**
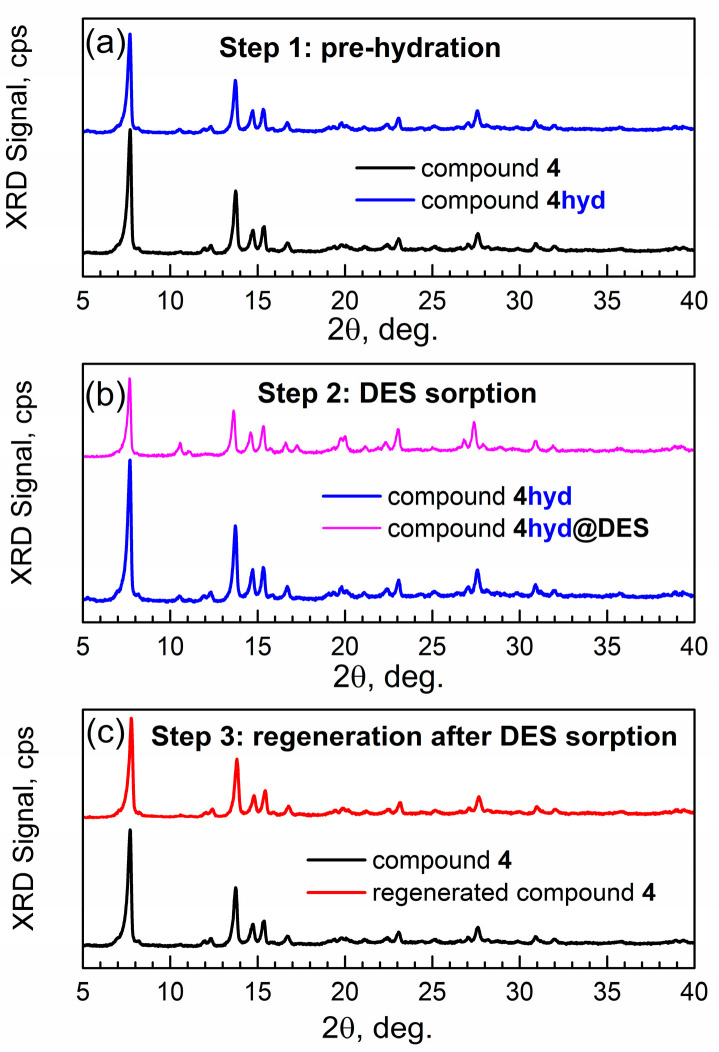
Powder XRD patterns of sorbent compound 4 and its adsorption complexes. (**a**) actAl-MOF-TCPPCu and hydAl-MOF-TCPPCu (binary adsorption complex). (**b**) hydAl-MOF-TCPPCu and DES-hydAl-MOF-TCPPCu (ternary adsorption complex). (**c**) actAl-MOF-TCPPCu and regAl-MOF-TCPPCu (regenerated ternary adsorption complex).

The latter was checked by powder XRD (Figure 9). In the first step of water pre-adsorption (Figure 9a), the XRD pattern remained unchanged upon the formation of the binary adsorption complex hydAl-MOF-TCPPCu. This indicates that this MOF is “rigid” upon the inclusion of water molecules, consistent with the observation of “rigidity” of the framework of related compound actAl-MOF-TCPPH_2_ during the sorption of DMF and water “guest” molecules [9].

In the second step, the binary complex was exposed to the saturated DES vapor under equilibrium (static) conditions. In Figure 9b, one can see the change in the XRD peak at ca. 10.7° after the sorption of DES. The sorption of DES affects the framework more (stronger interaction) than the sorption of water.

In the third step, the ternary adsorption complex was regenerated by heating under vacuum at 100 °C overnight. Figure 9c shows that the XRD patterns are identical for the regenerated ternary adsorption complex and actAl-MOF-TCPPCu, which indicates complete regeneration.

## 4. Conclusions

A new metal–organic framework, compound **4** Al-MOF-TCPPCu, readily sorbs the vapor of DES in dynamic conditions, as studied by in situ time-dependent ATR-FTIR spectroscopy, and interacts with DES via the hydroxy O-H, phenyl, and carboxylate COO^−^ groups. The kinetics of sorption obeys the Langmuir first-order model with the rate constant k(act) = 0.95 ± 0.05 min^−1^. Next, the pre-adsorption of water vapor at high RH = 75% yields the binary stoichiometric adsorption complex (Al-MOF-TCPPCu)_1.0_(H_2_O)_8.0_. It further interacts with DES vapor by the Langmuir first-order rate law from the beginning of the test, with a smaller rate constant k(hyd) = 0.16 ± 0.03 min^−1^. When under static (equilibrium) conditions, this binary adsorption complex adsorbs DES vapor, resulting in the ternary adsorption complex (Al-MOF-TCPPCu)_1.0_(H_2_O)_8.0_(DES)_3.8_. Finally, the ternary adsorption complex is facilely regenerated by gentle heating under vacuum.

## Data Availability

The original contributions presented in this study are included in the article/supplementary material. Further inquiries can be directed to the corresponding author.

## Supplementary Materials

The following supporting information can be downloaded at https://www.mdpi.com/article/10.3390/ma17246160/s1: Figure S1: Scheme of synthesis of compound **2** Al-MOF-TCPPH_2_ and its PSM reaction to the target compound **4** Al-MOF-TCPPCu. Figure S2: The UV-Vis calibration plot of copper ammonia complex for the spectrophotometric determination of copper. Figure S3: Digital photo of the pre-hydration chamber at controlled constant RH of air. Figure S4: The in situ ATR-FTIR spectrum of compound **4** actAl-MOF-TCPPCu. (a) High wavenumbers; (b) mid-IR; (c) low wavenumbers. Table S1: Major ATR-FTIR peaks of sorbent compound **4**. Figure S5: The ATR-FTIR spectrum of DES. (a) High wavenumbers; (b) mid-IR; (c) low wavenumbers. Table S2: Shifts of IR peaks of groups in activated compound **4** upon in situ interaction with DES. Figure S6: Changes in the prominent peaks of in situ time-dependent ATR-FTIR spectra of compound **4** in the flow of DES vapor. (a) C-H stretch of DES; (b) phenyl; (c) COO^−^; (d) deformation of μ-OH free group. Figure S7: Changes in IR peaks of groups in linker of compound **4**. (a) Pyrrole and deformation vibration of the porphyrin ring; (b) the phenyl group. Figure S8: Changes in the prominent peaks of in situ time-dependent ATR-FTIR spectra of compound **4hyd** in the flow of DES vapor. (a) C-H stretch of DES; (b) phenyl; (c) COO^−^; (d) deformation of μ-OH. Table S3: Shifts of IR peaks of groups in the binary complex upon in situ interaction with DES. Figure S9: Masses of specimens and steps. Pre-absorption of water vapor, the subsequent absorption of DES vapor, and sorbent regeneration.
