# Supplementary material for: Porphyrin-Based Aluminum Metal-Organic Framework with Copper: Pre-Adsorption of Water Vapor, Dynamic and Static Sorption of Diethyl Sulfide Vapor, and Sorbent Regeneration
Source: Materials (Basel). 2024 Dec 17;17(24):6160. doi: 10.3390/ma17246160 (PMC11677809; doi:10.3390/ma17246160)
Supplement: Supplementary file 1 [file materials-17-06160-s001.zip › materials-3336104-supplementary.pdf]

Supplementary Materials

Porphyrin-Based Aluminum Metal-Organic Framework with Copper: Pre-Adsorption of Water Vapor, Dynamic and Static Sorption of Diethyl Sulfide Vapor, and Sorbent Regeneration

Mohammad Shahwaz Ahmad and Alexander Samokhvalov *

Department of Chemistry, Morgan State University, 1700 East Cold Spring Lane, Baltimore, MD 21251, USA
* Correspondence: alexandr.samokhvalov@morgan.edu; Tel.: +1-443-885-4963

2. Materials and Methods

*2.1. Synthesis of actAl-MOF-TCPPCu (Compound **4**)*

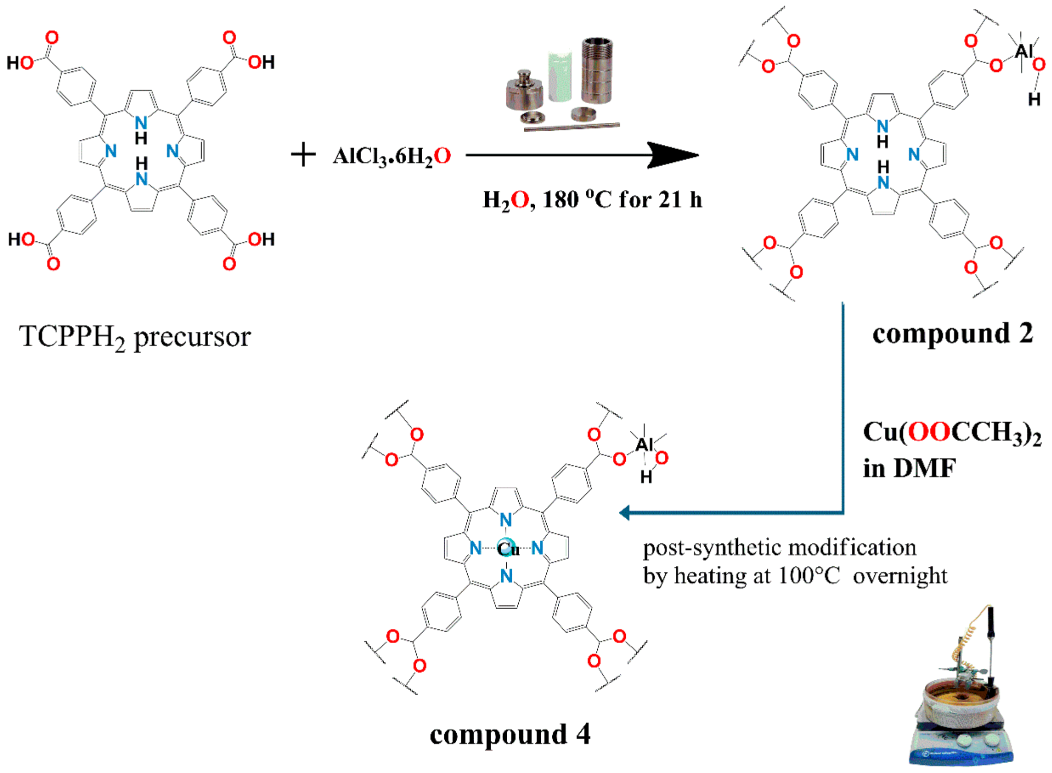

Figure S1. Scheme of synthesis of compound **2** Al-MOF-$TCPPH_2$ and its PSM reaction to the target compound **4** Al-MOF-TCPPCu.

*2.3. The Quantitative Spectrophotometric Determination of Copper in Compound **4***

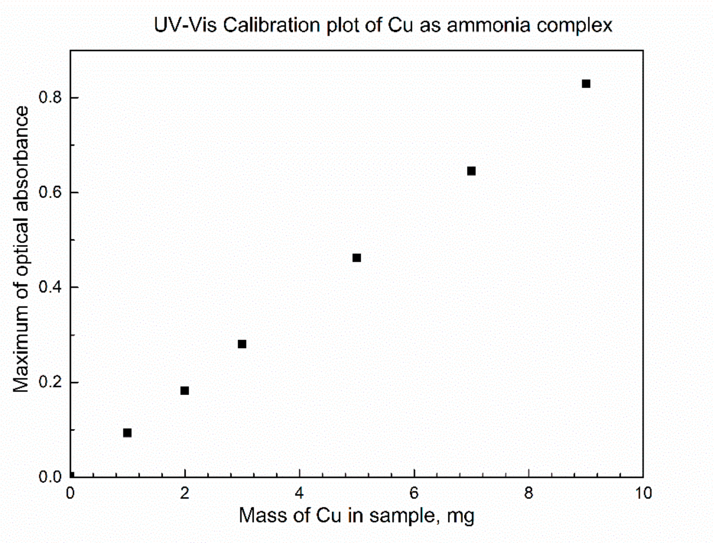

Figure S2. The UV-Vis calibration plot of copper ammonia complex for the spectrophotometric determination of copper.

*2.5. Pre-Hydration of Compound **4** actAl-MOF-TCPPCu with Water Vapor*

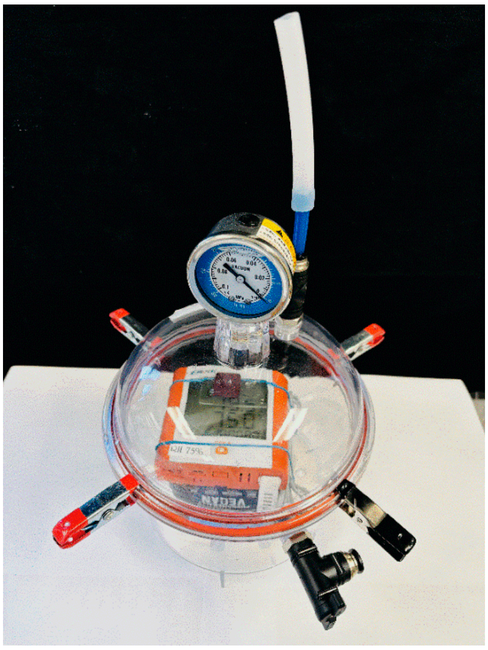

Figure S3. Digital photo of the pre-hydration chamber at controlled constant RH of air.

3. Results and Discussion

*3.1. Instrumental Analyses of Sorbent Compound **4** and Adsorbate DES*

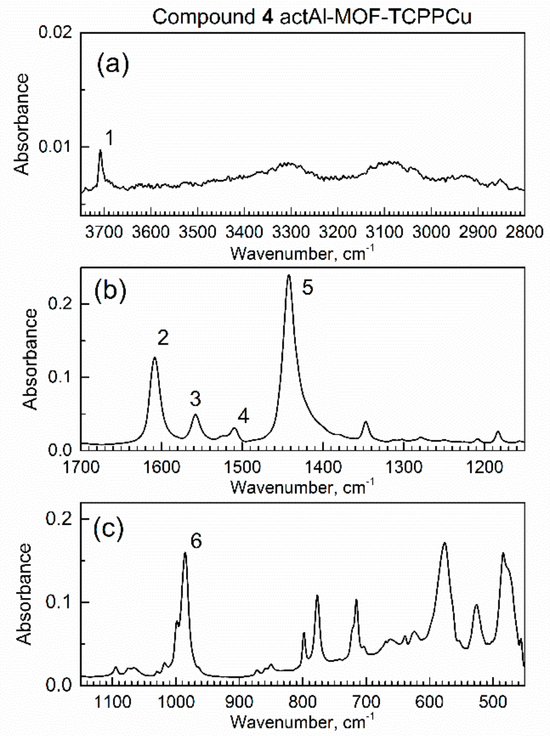

Figure S4. The in situ ATR-FTIR spectrum of compound **4** actAl-MOF-TCPPCu. a) High wavenumbers; b) mid-IR; c) low wavenumbers.

Table S1. Major ATR-FTIR peaks of sorbent compound **4**.

Peak number	Wavenumber (cm^{-1})	Functional group in compound **4**
1	3708 w	ν (O-H) free
2	1607	def (phenyl)
3	1556	ν_{asym} (COO^-)
4	1510	ν_{asym} (COO^-)
5	1442	ν_{sym} (COO^-)
6	985	def (μ-OH) free

ν = stretch; def = deformation; w = weak; μ = the O-H group connected to Al atom in MOF.

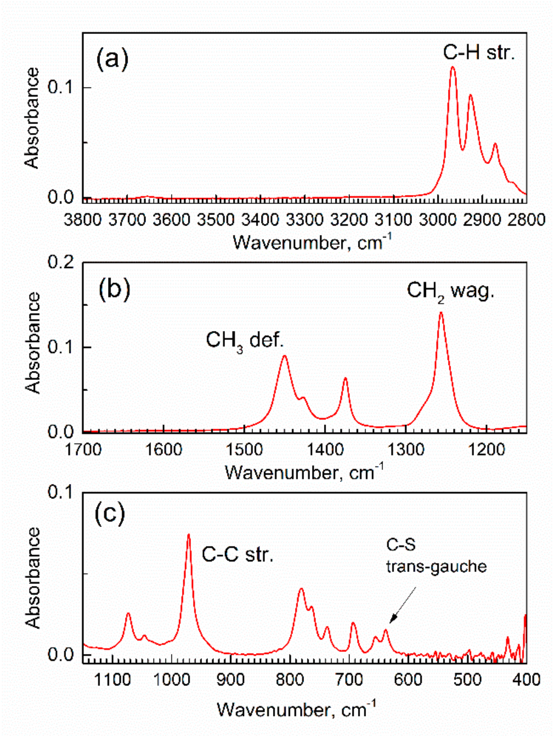

Figure S5. The ATR-FTIR spectrum of DES. a) High wavenumbers; b) mid-IR; c) low wavenumbers.

*3.2. The Progressive Sorption of DES Vapor by Activated Compound **4** Using In Situ Time-Dependent ATR-FTIR Spectroscopy*

Table S2. Shifts of IR peaks of groups in activated compound **4** upon in situ interaction with DES.

Group in sorbent	Peak center before sorption, cm^{-1}	Peak shift upon sorption of DES,	Bonding mechanism
Stretch / O-H	$\nu_1 = 3708$	$\Delta\nu_1 = -15$	the O-H
C=C / phenyl	$\nu_2 = 1607$	$\Delta\nu_2 = -3$	dispersive
asymm / COO^-	$\nu_3 = 1442$	$\Delta\nu_3 = -6$	polar bond

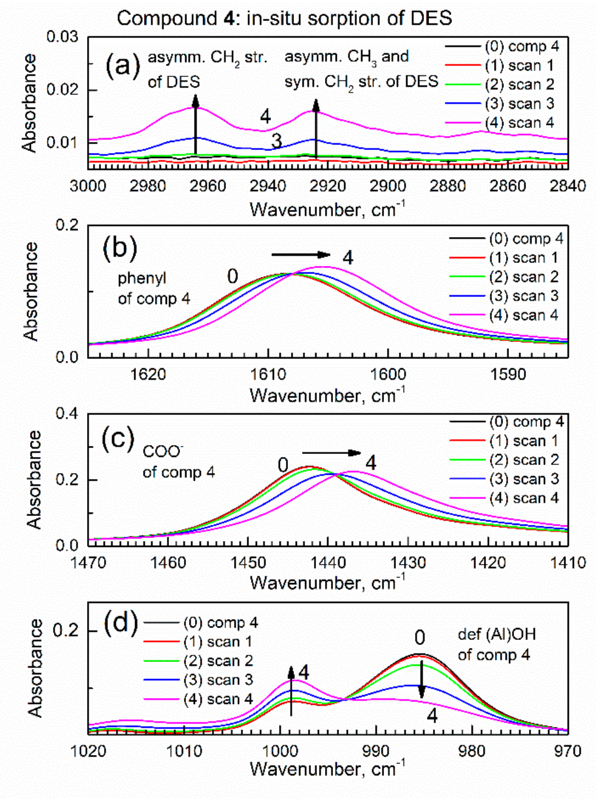

Figure S6. Changes in the prominent peaks of in situ time-dependent ATR-FTIR spectra of compound **4** in the flow of DES vapor. a) C-H stretch of DES; b) phenyl; c) COO^-; d) deformation of μ-OH free group.

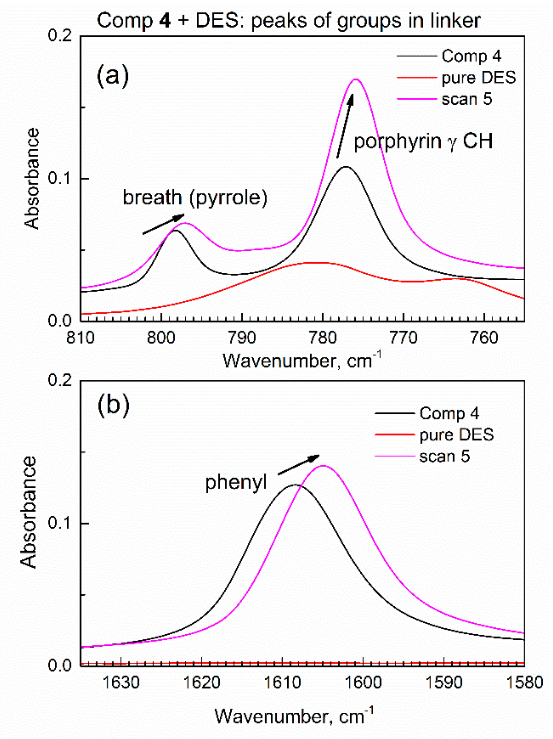

Figure S7. Changes in IR peaks of groups in linker of compound **4**. a) Pyrrole and deformation vibration of the porphyrin ring; b) the phenyl group.

*3.4. The Pre-Hydration of Compound **4**, Followed by Dynamic Sorption of DES Vapor by In Situ Time-Dependent ATR-FTIR Spectroscopy in a Controlled Atmosphere*

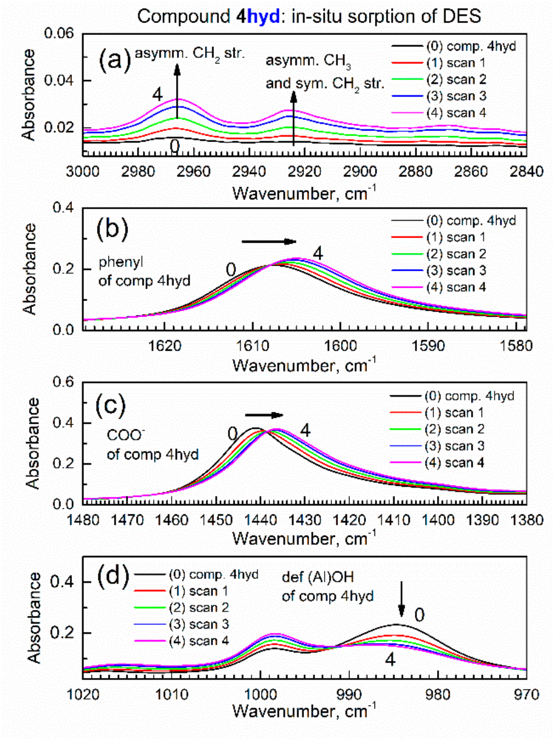

Figure S8. Changes in the prominent peaks of in situ time-dependent ATR-FTIR spectra of compound **4hyd** in the flow of DES vapor. a) C-H stretch of DES; b) phenyl; c) COO^-; d) deformation of μ-OH.

Table S3. Shifts of IR peaks of groups in the binary complex upon in situ interaction with DES.

Group in sorbent	Peak center before sorption, cm^{-1}	Peak shift upon sorption of DES, cm^{-1}	Bonding mechanism
Phenyl	$\nu_2 = 1608.5$	$\Delta\nu_2 = -4.0$	dispersive
COO^-	$\nu_3 = 1441.3$	$\Delta\nu_3 = -5.4$	polar bonding

*3.5. The Pre-Hydration of Compound **4**, Followed by the Static Sorption of DES Vapor and Sorbent Regeneration*

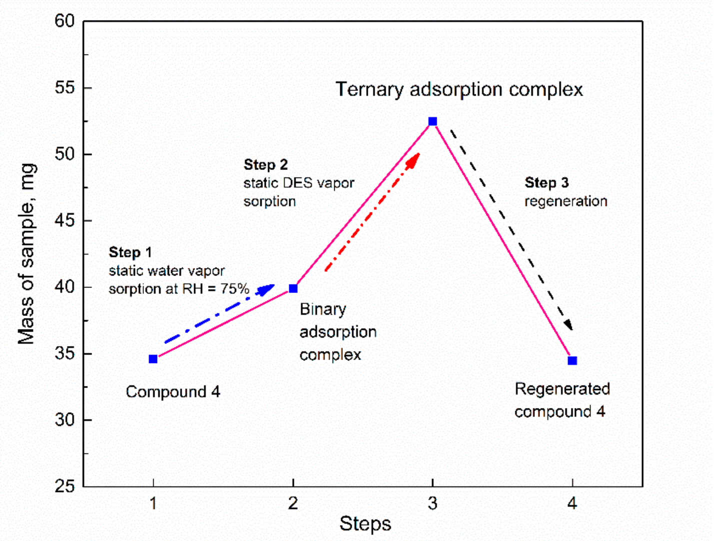

Figure S9. Masses of specimens and steps. Pre-absorption of water vapor, the subsequent absorption of DES vapor, and sorbent regeneration.
